# Why Use the Radial Artery? The Saphenous Vein is the Second Graft of Choice for CABG in Brazil

**DOI:** 10.21470/1678-9741-2019-0212

**Published:** 2019

**Authors:** Andrzej Loesch, Bruno Botelho Pinheiro, Michael Richard Dashwood

**Affiliations:** 1 Centre for Rheumatology, University College London Medical School, London, United Kingdom.; 2 Department of Cardiovascular Surgery, Hospital do Coração Anis Rassi, Goiânia, GO, Brazil.; 3 Surgical and Interventional Sciences, University College London Medical School, London, United Kingdom.

**Keywords:** Humans, Saphenous Vein, Radial Artery, Coronary Artery Bypass

## Abstract

The saphenous vein (SV) is the most commonly used conduit for coronary artery bypass surgery (CABG) and the second conduit of choice in Brazil and many other countries. The radial artery (RA) is suggested, by some, to be superior to SV grafts, although its use in the USA declined over a 10 year period. The patency of SV grafts (SVG) is improved when the vein is harvested with minimal trauma using the no-touch (NT) technique. This improved performance is due to the preservation of the outer pedicle surrounding the SV and reduction in vascular damage that occurs when using conventional techniques (CT) of harvesting. While the patency of NT SVGs has been shown superior to the RA at 36 months in one study, data from the RADIAL trial suggests the RA to be the superior conduit. When additional data using NT SVG is included in this trial the difference in risk of graft occlusion between the RA and SV grafts dissipates with there no longer being a significant difference in patency between conduits. The importance of preserving SV structure and the impact of NT harvesting on conduit choice for CABG patients are discussed in this short review.

**Table:** 

Abbreviations, acronyms & symbols	
CABG	= Coronary artery bypass grafting
CI	= Confidence interval
CT	= Conventional
LITA	= Left internal thoracic artery
M-H	= Mantel-Haenszel
NT	= No-touch
RA	= Radial artery
RADIAL	= Radial Artery Database International Alliance
RITA	= Right internal thoracic artery
SV	= Saphenous vein
SVG	= Saphenous vein graft

A recent article in the Brazilian Journal of Cardiovascular Surgery described an analysis of the profile, risk factors, and outcomes of patients undergoing coronary artery bypass grafting (CABG) in Brazil^[1]^. As in other countries, the left internal thoracic artery (LITA) is the conduit of choice and used in 91% of the cases, with 5.6% of cases using the right internal thoracic artery (RITA). The second graft of choice in Brazil is the saphenous vein (SV), being used in 84.1% of the cases, with the radial artery (RA) being used in only 1.1% of cases (Paez et al.^[1]^). The SV was introduced as a bypass conduit by Favaloro, 50 years ago^[2]^. This vein has certain properties making it particularly suitable for use as a graft since its characteristics are different from most veins. SV has a thick media and is subjected to pressure changes from ~10 to 80 mmHg associated with altered posture^[3]^, a situation ‘preconditioning’ this vessel when exposed to arterial conditions. The SV also has a number of practical advantages: it is expendable, since lower limb venous drainage can rely solely on the deep venous system, and its superficial position renders it easily accessible, facilitating its exposure at harvest^[2,4]^.

Interestingly, in a recent Expert Opinion article, “Additional arterial conduits in coronary artery bypass surgery: Finally coming of age”, Gaudino et al.^[5]^ acknowledge the important contribution of Favaloro when introducing SV as a bypass conduit. The authors then proceed to promote “… internal thoracic or radial arteries… as the ideal choice of conduits for revascularization”^[5]^. This statement is based on the Radial Artery Database International Alliance (RADIAL) project, an individual patient-level meta-analysis developed to adequately power a study to assess if RA has superior clinical outcomes compared with SV graft (SVG)^[6]^. The RA was originally introduced in the 1970s, but it was soon abandoned because of early graft failure^[7]^. However, the use of RA was revived about 20 years later after refining the harvesting, routine calcium channel blocker administration, and careful choice of coronary targets. The resurgence of RA as a conduit for CABG has led to a recent flurry of publications promoting it as the superior graft: reaching about 40 publications in the last five years, and rising rapidly^[5,6,8]^.

Despite these efforts to resurrect RA as the second graft of choice, a recent report of 10-year temporal trends of multi-arterial CABG showed a 64.8% decline (from 10.5% to 3.7%) in its use in the United States of America between 2004 and 2014^[9]^. While RA use in these patients declined, the use of SV remained fairly constant over the same time period. Clearly, the very recent data from the Brazilian BYPASS Registry shows that the preferred second conduit of choice for CABG in Brazil is SV^[1]^. Furthermore, many centres in Brazil use the no-touch (NT) technique of harvesting SV that was introduced over 20 years ago^[10]^, which provides a superior graft patency compared with conventional SV grafts at up to 16 years and is comparable to the LITA’s patency^[11,12]^.

In contrast to data from the RADIAL studies, NT SV grafts were shown to be superior to RA grafts (*P*=0.01) at a mean of 36 months postoperatively^[13]^. Why should there be this discrepancy? It appears that the RADIAL studies compared ‘conventional’ (CT) SVs that were harvested following Favaloro’s method where they were ‘injured’ during removal. It is generally accepted that SVs harvested in this fashion provide grafts with a failure rate of 50% within 10 years^[14,15]^. The NT SV vs. RA data from Dreifaldt et al.^[13]^ was excluded from the analysis by the RADIAL group^[5,8]^. Also, the study by Song et al.^[16]^, which was included in the RADIAL group analysis, employed NT harvesting and had RA with numerically lower patency than NT SV. If the angiographic patency data of the five trials with protocol-driven angiography are supplemented with the data from the Örebro group, the difference in risk of graft occlusion between RA and SV grafts dissipates (Figure 1)^[17]^. A recent Feature Expert Opinion by An et al.^[18]^ again provides evidence to support the use of RA compared to the SV graft for CABG, citing the study recently published in the New England Journal of Medicine by Gaudino et al.^[8]^. Despite the assertion that “use of radial-artery grafts resulted in a significantly lower rate of major adverse cardiac events and a better patency rate (than the SV) at a postoperative follow-up of 5 years”, it appears that not all are impressed with this data. This is particularly evident in a recent Editorial Commentary remarking on the “meta-analysis of (RA *vs*. SV) trials that are 6 to 15 years old”. Here it is proposed that “Gaudino and colleagues’ well-executed patient-level meta-analysis did a fine job of turning a sow’s ear of underpowered randomized controlled trials into a silk purse with a few suggestive *P* values”^[19]^. This author not only considers the adverse effect that RA removal may have on the ability of surgeons themselves undergoing CABG to continue operating but amusingly requests, “please do not use my RA - I want to be able to play my piano after I am forced to retire”. In addition to the potential problems described above when using the RA^[20]^, there is the fact that it is prone to spasm (especially if the target coronary artery has a < 90% stenosis) and there are also occasions when this vessel is unavailable or unsuitable for use as a graft, including patients with chronic renal disease.

**Fig. 1:**
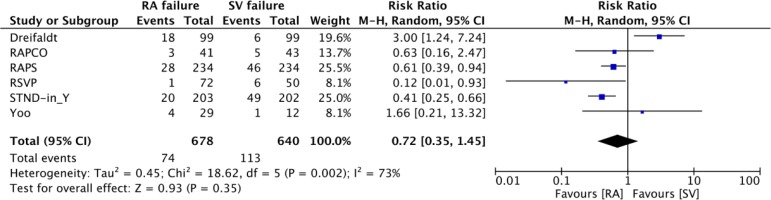
Forest plot of comparison: saphenous vein (SV) vs. radial artery (RA) patency. Data pooling was based on six randomized controlled trials with protocol-driven angiography comparing SV and RA patency. No significant difference in risk of graft failure was observed between SV and RA grafts. CI=confidence interval; M-H=Mantel-Haenszel. (From Kopjar et al. ^[17]^).

We believe it is important to consider the condition of the SVs used for coronary revascularization since it seems that, too often, a ‘conduit’ is considered merely a connecting tube or pipe rather than a ‘viable graft’. Why are arterial conduits usually harvested ‘non-injured’, with pedicle intact, whereas SVs are ‘injured’ at harvesting when the pedicle is removed? (Figure 2). Many of the repair processes following this injury are involved in the pathophysiology of SV graft failure. The aforementioned RADIAL trials compared ‘intact’ RA with ‘damaged’ SV grafts. This is supported by the appearance of CT SV and NT SV grafts at harvesting and post-mortem in trials where NT SV grafts were superior, remaining patent after 16 years^[11,12]^ (Figure 2). The vascular trauma and distension that occurs at CT SV harvesting has a harmful effect on various structures and factors beneficial to the preservation of a healthy graft such as the endothelium/nitric oxide axis, adipocyte-derived relaxing factors, the vasa vasorum, and the mechanical and other properties of perivascular fat^[21]^. A number of strategies have been introduced in an effort to improve CT SV graft performance, ranging from gene targeting and the application of fibrin glue to the fitting of external stents to provide mechanical support for the graft^[21,22]^. Why should this be necessary? Such strategies merely aim to repair the damage inflicted when using CT SV harvesting.

**Fig. 2:**
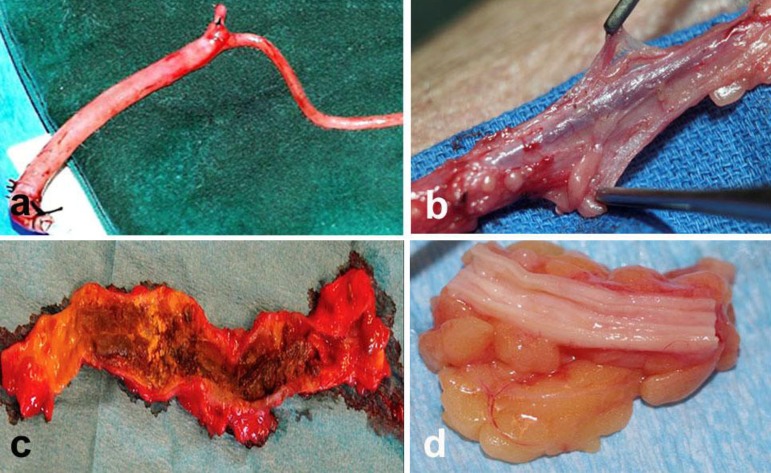
Saphenous vein grafts (SVG) at harvesting and post-mortem. a. Conventional SVG stripped of surrounding tissue and distended to overcome constriction (to the right of the branch). b. No-touch SVG with perivascular fat, adventitia, and vasa vasorum intact. c. Post-mortem conventional SVG at 8 years after CABG shows signs of considerable necrotic and friable tissue, as well as a diffuse atherosclerotic process. d. Post-mortem no-touch SVG 18 years after surgery where the atherosclerotic process is much reduced when compared with conventional SVGs. Images modified from: a. Souza et al. ^[11]^ 2006: b-d. Samano et al. ^[12]^ 2015.

The NT technique has been widely recognised and was recommended almost 20 years ago as a method of preserving SV integrity and improving graft performance^[23]^_._ This technique is routinely used in many centres in Brazil and in many other countries including Japan, Russia, China, Croatia, Norway, and Korea. While there may be a difference of opinion regarding the preferred second conduit of choice, a growing number of surgeons have adopted NT SV in preference to RA.

**Table:** 

Author's roles & responsibilities	
AL	Substantial contributions to the conception or design of the work; drafting the work or revising it critically for important intellectual content; agreement to be accountable for all aspects of the work in ensuring that questions related to the accuracy or integrity of any part of the work are appropriately investigated and resolved; final approval of the version to be published
BBP	Substantial contributions to the conception or design of the work; drafting the work or revising it critically for important intellectual content; agreement to be accountable for all aspects of the work in ensuring that questions related to the accuracy or integrity of any part of the work are appropriately investigated and resolved; final approval of the version to be published
MRD	Substantial contributions to the conception or design of the work; drafting the work or revising it critically for important intellectual content; agreement to be accountable for all aspects of the work in ensuring that questions related to the accuracy or integrity of any part of the work are appropriately investigated and resolved; final approval of the version to be published
